# Evaluating the effectiveness of organisational-level strategies with or without an activity tracker to reduce office workers’ sitting time: a cluster-randomised trial

**DOI:** 10.1186/s12966-016-0441-3

**Published:** 2016-11-04

**Authors:** C. L. Brakenridge, B. S. Fjeldsoe, D. C. Young, E. A. H. Winkler, D. W. Dunstan, L. M. Straker, G. N. Healy

**Affiliations:** 1 School of Public Health, The University of Queensland, Brisbane, Australia; 2 Lendlease, Sydney, Australia; 3 Baker IDI Heart and Diabetes Institute, Melbourne, Australia; 4 School of Public Health and Preventive Medicine, Monash University, Melbourne, Australia; 5 School of Exercise and Nutrition Sciences, Deakin University, Melbourne, Australia; 6 Department of Medicine, Monash University, Melbourne, Australia; 7 School of Sport Science, Exercise and Health, The University of Western Australia, Perth, Australia; 8 Mary MacKillop Institute for Health Research, Australian Catholic University, Melbourne, Australia; 9 School of Physiotherapy and Exercise Science, Curtin University, Perth, Australia

**Keywords:** Wearable device, Self-monitoring, Sedentary, Office workers, Light intensity activity, Ecological model, Workplace, Trial, Objective, Activity monitor

## Abstract

**Background:**

Office workers engage in high levels of sitting time. Effective, context-specific, and scalable strategies are needed to support widespread sitting reduction. This study aimed to evaluate organisational-support strategies alone or in combination with an activity tracker to reduce sitting in office workers.

**Methods:**

From one organisation, 153 desk-based office workers were cluster-randomised (by team) to organisational support only (e.g., manager support, emails; ‘Group ORG’, 9 teams, 87 participants), or organisational support plus LUMOback activity tracker (‘Group ORG + Tracker’, 9 teams, 66 participants). The waist-worn tracker provided real-time feedback and prompts on sitting and posture. ActivPAL3 monitors were used to ascertain primary outcomes (sitting time during work- and overall hours) and other activity outcomes: prolonged sitting time (≥30 min bouts), time between sitting bouts, standing time, stepping time, and number of steps. Health and work outcomes were assessed by questionnaire. Changes within each group (three- and 12 months) and differences between groups were analysed by linear mixed models. Missing data were multiply imputed.

**Results:**

At baseline, participants (46 % women, 23–58 years) spent (mean ± SD) 74.3 ± 9.7 % of their workday sitting, 17.5 ± 8.3 % standing and 8.1 ± 2.7 % stepping. Significant (*p* < 0.05) reductions in sitting time (both work and overall) were observed within both groups, but only at 12 months. For secondary activity outcomes, Group ORG significantly improved in work prolonged sitting, time between sitting bouts and standing time, and overall prolonged sitting time (12 months), and in overall standing time (three- and 12 months); while Group ORG + Tracker, significantly improved in work prolonged sitting, standing, stepping and overall standing time (12 months). Adjusted for confounders, the only significant between-group differences were a greater stepping time and step count for Group ORG + Tracker relative to Group ORG (+20.6 min/16 h day, 95 % CI: 3.1, 38.1, *p* = 0.021; +846.5steps/16 h day, 95 % CI: 67.8, 1625.2, *p* = 0.033) at 12 months. Observed changes in health and work outcomes were small and not statistically significant.

**Conclusions:**

Organisational-support strategies with or without an activity tracker resulted in improvements in sitting, prolonged sitting and standing; adding a tracker enhanced stepping changes. Improvements were most evident at 12 months, suggesting the organisational-support strategies may have taken time to embed within the organisation.

**Trial registration:**

Australian New Zealand Clinical Trial Registry: ACTRN12614000252617. Registered 10 March 2014.

**Electronic supplementary material:**

The online version of this article (doi:10.1186/s12966-016-0441-3) contains supplementary material, which is available to authorized users.

## Background

The workplace has been identified as a key target area for health promotion [1] and offers an environment that enables reach to large numbers of people simultaneously. Furthermore, it is a setting where adults spend much of their waking day [2, 3], enabling multiple opportunities for intervention, and for any changes to potentially have a considerable impact.

An emerging workplace health issue is excessive sitting [4]. Office workers spend, on average, three-quarters of their work hours sitting [5]. Much of this sitting time is accrued in prolonged, unbroken bouts of at least 30 min at a time [5]; a pattern that may be particularly detrimental for cardiovascular [6–8] and musculoskeletal [9] health. In light of the health impacts of too much sitting, various strategies have now been implemented in the workplace setting aimed at reducing prolonged sitting time both in, and outside, the workplace [10].

Support from the organisation for behaviour change is considered a core element for both program implementation [11] and long-term success [12]. Strategies that facilitate organisational support for programs include: having senior and mid-level management support; management participation in programs; and, having a dedicated wellness ‘champion’ onsite to gain management endorsement and promote the programs [13, 14]. Many workplace sitting interventions that implement organisational-support strategies do so in conjunction with strategies targeting environmental (e.g., through the use of sit-stand workstations) and individual (e.g., through health coaching) influences on behaviour change [15–17]. Although this multi-component approach is highly effective [15–17], it is resource intensive. Practically, awareness raising techniques (such as information sessions and posters) and visible support of the program by the organisation, are likely to be some of the initial strategies utilised prior to investing in sit-stand workstations and/or health coaching. As such, it is important to evaluate the potential effect of these minimally intensive approaches on short and long-term behaviour change.

Similarly, the emergence and rapid uptake of sophisticated consumer-targeted wearable technology provides an opportunity for employees to measure and track their own activity levels beyond just basic pedometer step counts [18, 19]. Whilst much evidence suggests that pedometers can successfully increase activity and reduce sitting in the workplace [20–22], only three studies to date have evaluated the impact of more advanced consumer-targeted activity trackers in an office workplace setting [23–25]. In all cases evaluation was on activity outcomes only, with mixed results. Despite the use of consumer-targeted trackers in corporate wellness plans [18, 19] and challenges [26], and their expected increased use in the workforce [19], the extent to which such devices add to any organisational-level approaches to reduce sitting time has not yet been evaluated.

The primary aim of this study was to assess the short-term (three-month) effectiveness of two interventions (organisational support with and without an activity tracker) for improving work and overall sitting time (primary outcomes) in office workers. This paper also evaluates the long-term (12-month) effectiveness on these primary outcomes, as well as both the short and long-term effectiveness of the two interventions on secondary outcomes (other activities, work performance- and health-related outcomes). In addition to within group changes, the relative effectiveness of the two interventions for improving activity in the short and long term was also assessed.

## Methods

An organisational-support intervention (Group ORG) and the identical intervention but with the addition of an activity tracker (Group ORG + Tracker) were evaluated in a clustered-randomised trial (*Stand Up Lendlease)*, with work teams as the unit of clustering. Data collection occurred at baseline, three- and 12 months. The trial protocol, including a detailed description of the measures, has been published [27]. The trial was approved by the University of Queensland Behavioural and Social Sciences Ethical Review Committee (approval number: 2014000089) and was prospectively registered with the Australian New Zealand Clinical Trials Registry (registration number: ACTRN12614000252617). This study complies with the CONSORT Extension for Cluster Trials Checklist and the TIDieR Checklist (Additional file 1).

### Participant recruitment and eligibility

An international property and infrastructure group, Lendlease, volunteered to take part in the study. Teams of employees were approached to participate from offices at two Australian capital cities: Sydney (Head Office, one site, ‘location A’) and Brisbane (three closely located sites, ‘location B’). To be eligible teams had to work at the relevant location (A or B) or work near to and regularly visit the Head Office (location A). There were approximately 1525 employees across these two locations, with the majority (1200) of employees at location A. Recruitment and baseline assessments occurred between March and April 2014.

The workplace champion (see below) approached team managers (including himself) to participate, obtained informed consent and established team eligibility. Team members were invited to attend an information session, during which individual staff eligibility was confirmed, informed written consent was obtained, and the baseline assessment was undertaken.

Full eligibility criteria have been reported previously [27]. The minimum proportion of full-time equivalent (FTE) work was modified from initially being 0.6FTE in the protocol [27] to 0.5FTE (i.e., 50 % of full-time work hours) as the research team considered that this still constituted sufficient time at the workplace to benefit from the intervention. Initial consent was only for baseline and three-month assessments, participants were invited to re-consent for the 12-month assessment.

### Randomisation

Randomisation occurred after the final list of team managers for each location had been obtained and prior to the staff information session and baseline assessment. A university staff member not involved in the study randomised teams by strata (location B/small location A teams/large location A teams) to either Group ORG or Group ORG + Tracker, using a randomisation website [28]. The six smallest of the participating teams for location A were classified as ‘small’. A research team member then applied the randomisation schedule to the list of teams and informed the champion of the allocation. Neither the research team nor participants were blinded to participants’ randomisation status.

### Interventions

#### Organisational support development and strategies

The Lendlease Head of Workplace Wellbeing (DCY) volunteered as the study’s workplace champion. His normal job role entailed involvement in several workplace health initiatives, and providing workplace health-related presentations to the wider organisation. Consequently, he was ideally suited to deliver the intervention in a manner that was sensitive and relevant to the organisation’s needs and sustainable within the organisation. The champion was given no further health promotion training prior to the study. The workplace champion was responsible for recruitment, delivery of the intervention, distribution and collection of equipment, and communications with the participants regarding their study participation. The researcher involvement included technology support with the activity tracker and evaluating the intervention.

The initial discussions between the research team and the champion were in regards to the feasibility of the study and the resources required. The champion then gained senior management support for the study through discussions with the CEO and other senior executives. Once this approval was attained, the research team provided the champion with a range of strategies that have been successfully implemented as part of the broader *Stand Up Australia* program of research [29]. The champion then chose which strategies were deemed to be suitable for the organisation (see Table 1).

**Table 1:** Intervention strategies employed during the first three months of the study

Strategy	Week 1	Weeks 3–4	Week 6	Weeks 7–8	Weeks 9–10	Weeks 11–13
Organisational	Introductory email to announce program from champion + workplace summary activity data + links to relevant news articles Information booklet including: • Health impacts of sitting • Tips to ‘Stand Up, Sit Less and Move More’	First tip email from champion^a^ Tip: have a standing or walking meeting	Second tip email^b^ Tip: walking meetings increase creativity	Third tip email Focus on increasing step count + Table of step count classifications compared to baseline average^c^ + Walking step count guide of locations around Sydney office^d^ Tip: walking meetings increase step count	Fourth tip email Focus on standing more and sitting less outside of work Tips: • Walk during commercial breaks • Do household chores while watching TV • Stand to read newspaper • Hand wash car • Move around house when checking emails and texts on phone • Take your coffee standing • Stand up every time there is a goal in the World Cup • Stand up while commuting to work	Fifth tip email Focus on using the stairs Tip: take the stairs instead of the lift
Individual	Activity tracker to Group ORG + Tracker participants	Baseline activity monitor feedback sent				

^a^sent in week 1 for location B; ^b^sent in week 4 for location B; ^c^step count classifications from Tudor-Locke 2004 [61]; ^d^location A only

The first implemented strategy was an information booklet emailed in week 1 by the champion to all participating staff. The booklet contained background information on sitting and health implications, an introduction to the *Stand Up Lendlease* program, and recommendations and tips to ‘Stand Up, Sit Less and Move More’. The information booklet was sent out with an introductory email that had a preliminary summary of the averaged activity monitor data from the baseline assessment (based on the first 62 completed and processed assessments; see further details below) and additional web links about the health effects of prolonged sitting.

The next strategy involved five fortnightly emails developed in a partnership between the research team and the champion, and sent to participants by the champion (Table 1). Based on the *Stand Up Australia* email template [30], the emails were modified by the champion to include his chosen activity-promoting tips, comments from participants or managers, images of participants taking part in the ‘Stand Up, Sit Less, Move More’ message and the organisation’s branding (see Additional file 2). Ideas for tips came from the Heart Foundation of Australia’s tip sheet [31], and included tips to have standing and walking meetings (see Table 1).

To visibly demonstrate support for the program and its messages, senior executives took part in the baseline assessment and received the five emails. Their participation in the study was communicated to participants by the champion. During the 12-month intervention period the champion also presented at least 10 workplace presentations as part of his Workplace Wellbeing role and continued to have informal discussions with managers about their team’s sedentary work practices. Individual baseline, three- and 12-month feedback from the activity monitor assessments was also emailed to participants by the research team.

#### Activity tracker

Participants in Group ORG + Tracker also received a LUMOback activity tracker (LUMO Bodytech, Mountain View, CA, USA) along with an instruction booklet in study week 1. The LUMOback was worn as a belt and synced with the associated smartphone mobile application (app) which provided feedback on sitting, standing, stepping, sitting breaks, posture and sleep. The LUMOback was chosen by the research team as it is one of the few activity trackers to measure and target sitting time in addition to activity. LUMOback usage was self-directed rather than prescribed by the protocol (i.e., participants could wear the device as much or as little as they liked) because a key study aim (not addressed in this paper) was to evaluate the device’s acceptability and usage. Data recorded by the LUMOback was requested from LUMO Bodytech by the research team every two weeks to measure device usage. Non-users were contacted between weeks three and 10 by phone (CLB), email (CLB), or face to face (DCY) to discuss any trouble in using or setting up the LUMOback, and provide support as appropriate. Participants were free to keep the LUMOback.

### Data collection

Data on activity during work hours and overall (work and non-work time combined) were collected at baseline, three- and 12 months, using the activPAL3^TM^ (PAL Technologies Ltd., Glasgow, Scotland, UK; software version ≥6.4.1). The activPAL provides highly accurate and responsive measures of sitting/lying (referred to as sitting), standing, and stepping time [32, 33] and sitting accumulation patterns [33]. Assessment dates for each stage are presented in Additional file 3. At each assessment, participants were asked to wear the activPAL3 on the dominant thigh for seven consecutive days (24 h/day). Attendees of the information session received an in-person demonstration on how to attach the waterproofed activPAL using hypoallergenic adhesive Hypafix, along with written instructions. Non-attendees and all participants at the follow-up assessments were provided with written instructions only. Participants were asked to report in an electronic diary when they started and finished work, went to bed and woke up, and removed and re-attached the monitor.

An online questionnaire (hosted by LimeService [34]) was sent to participants after their activPAL assessment. The questionnaires collected data on health outcomes and work outcomes, socio-demographic data (at baseline only) and intervention fidelity and strategy use data. Objective (*n* = 107, 90.7 %) and self-report (*n* = 11, 9.3 %) height and weight data were collected at baseline from which body mass index (BMI, kg/m^2^) was calculated. Use of the LUMOback was determined through the data received from LUMO Bodytech (a valid day was counted as one hour or more of wear) or participant self-report (questionnaire or telephone interview) if data were missing or less than one hour per day.

#### Activity outcomes

The average time per day spent sitting during work hours and overall were the primary outcomes. The other activity outcomes, assessed during work hours and overall were: the average time per day spent in prolonged sitting bouts (sitting time accrued in continuous bouts of 30 min or more), standing, and stepping; the number of steps per day; and, the average time period between sitting bouts. This latter measure is a sensitive and responsive metric [35] of sitting or sedentary time accumulation.

A customised SAS program (version ≥9.3) was used to extract the activity data from the activPAL events files, limited to key diary-reported periods (awake, at work, wearing the monitor, and on valid days). The alignment of the diary and monitor data, and valid day definitions are described elsewhere [27]. Total time or steps per day were calculated then averaged over the valid days, and normalised to a 16-h waking day or 10-h workday (which were about average for this sample). Average time between sitting bouts was calculated as the mean duration of the upright periods between sitting bouts, using the maximum likelihood estimate of the mean for a log-normal distribution [35].

#### Work and health variables

Work-related outcomes were: job performance score (the mean of a 9-item, self-rated job performance scale, 1–10 scale) [36]; job control score (single item, 1–10 scale), and work satisfaction (mean of four items, 1–10 scale) as per the Health and Work Questionnaire [37] whereby higher scores indicate respectively higher self-rated job performance, more job control and more work satisfaction. Health-related outcomes were an overall stress score from the Health and Work Questionnaire (single item, 1–10 scale; higher scores indicate more stress) [37], and physical and mental health quality of life assessed by the Short Form (SF)-12 version 1 (12 items, 0–100 scale; higher scores indicate better quality of life) [38]. All of these work and health outcomes were considered as secondary outcomes. Musculoskeletal symptoms present at baseline (in the upper body, back, and lower extremities) over the last one month were assessed using the Nordic Musculoskeletal Questionnaire [39] and were considered as potential confounders.

#### Adverse events

Adverse events related to study participation were collected in the three- and 12 month questionnaires for both intervention groups. In addition, a specific question asked about adverse events relating to the LUMOback for Group ORG + Tracker. Adverse events were also recorded when a participant reported the event as a reason for not using the LUMOback, or, for either group, withdrawal, declining re-consent or being otherwise unable to take part in an assessment.

### Sample size

The sample size calculation is reported in detail elsewhere [27]. A sample size of 150 (18 clusters), with an anticipated 30 % attrition, and strong clustering (Intra-cluster Correlation Coefficient, ICC = 0.1; design effect = 1.48) and 5 % significance (no multiple comparison adjustment) was anticipated to provide 90 % power to detect changes equivalent to the minimum differences of interest (MDIs) for the primary outcomes, and to provide minimum detectable differences between groups for sitting of 50 min/day. This study was not powered *a priori* on health and work outcomes or long-term changes (12 months). MDIs were 45 min/day for sitting and prolonged sitting, 30 min/day for standing and 15 min/day for stepping. MDIs for step counts, average time between sitting bouts, work and health outcomes were set at one third of a standard deviation.

### Statistical methods

Analyses were conducted in SPSS Statistics version 22 (IBM Corp, Armonk, NY) and Stata version 13 (StataCorp LP, College Station, TX) with statistical significance set at *p* < 0.05, two tailed. Missing data exceeded the levels (e.g., 5 % or 10 %) at which results can be unbiased despite data not meeting the missing completely at random assumption [40, 41]. Missing data were handled by multiple imputation, minimising biases and preserving power [42], and providing intention-to-treat estimates that are consistent with CONSORT recommendations [43]. Chained equations, specifying truncated regression whenever possible to keep imputations within the natural bounds of the data [44] were used, with m = 70 imputations for activity outcomes and m = 80 imputations for work and health outcomes, based on the largest requirement according to fraction of missing information (m ≥ 100 × FMI) or percentage of missing information criteria [42]. Imputation models included all variables in the relevant analysis, age, sex, and additional predictors of missing data that were significant at *p* < 0.2 (Additional file 4).

Within-group changes were assessed using linear mixed models that account for repeated measures (baseline, 3 months, 12 months) and clustering (random intercept for cluster). Between-group differences were assessed using mixed models that accounted for repeated measures and clustering, and adjusted for baseline values and potential confounders. Potential confounders were selected using backwards elimination with *p* < 0.2 for retention (see Additional file 5). Models were checked for: convergence, misspecification problems, and that imputed values resembled observed data. From these models, we report changes or differences between groups with 95 % confidence intervals as estimated by comparisons of marginal means. Completers analyses are reported as a sensitivity analysis in Additional file 6. Unadjusted analyses are reported in Additional file 7.

## Results

### Participant characteristics

Baseline characteristics according to intervention group are presented in Table 2. Group ORG had a higher proportion of males, senior leaders and overweight participants, had less managers and reported more lower-extremity musculoskeletal problems than Group ORG + Tracker. Baseline activity levels were comparable: for Group ORG, the mean time (±SD) spent sitting, standing and stepping during work hours was 73.5 ± 9.9 %, 18.2 ± 8.1 %, and 8.3 ± 2.8 % respectively. The corresponding figures for Group ORG + Tracker were 75.5 ± 9.3 %, 16.7 ± 8.5 % and 7.8 ± 2.5 %. Team sizes ranged from three to 14; further details of characteristics by team are included in Additional file 8. ICCs for overall and work hour activity at baseline ranged between 0.027 (95 % CI: 0.001, 0.514) and 0.080 (95 % CI: 0.016, 0.320) (see Additional file 8).

**Table 2:** Baseline characteristics by group and overall

	Group ORG	Group ORG + Tracker	All participants
	*n* = 87^a^	*n* = 66^a^	*n* = 153^a^
Male	60 % (52)	47 % (31)	54 % (83)
Age, years	40.0 ± 8.0	37.6 ± 7.8	38.9 ± 8.0
BMI, kg/m^2^	25.0 ± 3.4	24.1 ± 3.4	24.6 ± 3.4
Normal weight	54 % (37^b^)	74 % (37)	63 % (74)
Overweight	37 % (25)	16 % (8)	28 % (33)
Obese	9 % (6)	10 % (5)	9 % (11)
University education	83 % (67)	86 % (54)	84 % (121)
1.0 Full-time equivalent	95 % (77)	92 % (59)	94 % (136)
*Job category*			
Manager	52 % (45)	64 % (42)	57 % (87)
Senior leader	16 % (14)	6 % (4)	12 % (18)
Other	32 % (28)	30 % (20)	31 % (48)
Smoker	10 % (8)	9 % (5)	10 % (13)
Weekday work hours/day^c^	9.9 ± 1.2	9.6 ± 1.0	9.8 ± 1.1
*Musculoskeletal*			
Upper body problems^d^	62 % (48)	64 % (37)	63 % (85)
Back problems^d^	55 % (42)	59 % (34)	56 % (76)
Lower extremity problems^d^	38 % (29)	26 % (15)	33 % (44)
Activity variables			
*Work hours*			
Sitting (min/10 h workday)	440.8 ± 59.6	453.0 ± 55.9	446.0 ± 58.2
Prolonged sitting (min/10 h workday)	246.3 ± 81.6	254.8 ± 89.8	249.9 ± 85.0
Time between sitting bouts (min)	5.9 ± 2.8	5.1 ± 4.5	5.6 ± 3.6
Standing (min/10 h workday)	109.1 ± 48.5	100.2 ± 50.8	105.3 ± 49.5
Stepping (min/10 h workday)	50.1 ± 16.6	46.8 ± 15.1	48.7 ± 16.0
Step count (number of steps/10 h workday)	2350.0 ± 791.5	2201.2 ± 748.3	2286.8 ± 774.3
*Overall hours*			
Sitting (min/16 h day)	618.4 ± 71.7	627.0 ± 65.2	622.1 ± 68.9
Prolonged sitting (min/16 h day)	321.8 ± 89.8	333.3 ± 96.9	326.7 ± 92.8
Time between sitting bouts (min)	7.3 ± 2.4	6.6 ± 1.9	7.0 ± 2.2
Standing (min/16 h day)	229.8 ± 57.8	216.9 ± 56.3	224.3 ± 57.4
Stepping (min/16 h day)	111.8 ± 29.0	116.1 ± 26.3	113.7 ± 27.9
Step count (number of steps/16 h day)	4886.7 ± 1444.3	5160.0 ± 1410.0	5004.0 ± 1431.3
Workplace variables			
Job performance scale^e^	7.5 ± 0.9	7.7 ± 0.9	7.6 ± 0.9
Job control^e^	6.8 ± 1.8	7.0 ± 1.9	6.8 ± 1.8
Work satisfaction^e^	6.3 ± 1.4	6.6 ± 1.6	6.4 ± 1.5
Health variables			
Stress^e^	6.6 ± 2.1	6.2 ± 2.5	6.5 ± 2.3
Physical health quality of life^f^	50.9 ± 8.0	51.8 ± 6.8	51.3 ± 7.5
Mental health quality of life^f^	44.0 ± 11.9	46.1 ± 10.3	44.9 ± 11.2

Data are % (n) or mean ± SD

^a^All *n* = 87 Group ORG, *n* = 66 Group ORG + Tracker (sex and job category); *n* = 86 Group ORG (weekday work hours/day), *n* = 81 Group ORG (FTE, education), *n* = 80 Group ORG (age), *n* = 64 Group ORG + Tracker (FTE), *n* = 63 Group ORG + Tracker (weekday work hours/day, age, education); *n* = 77 Group ORG, *n* = 58 Group ORG + Tracker (musculoskeletal items; smoking status); *n* = 74 Group ORG, *n* = 55 Group ORG + Tracker (job control); *n* = 72 Group ORG, *n* = 56 Group ORG + Tracker (quality of life); *n* = 75 Group ORG, *n* = 56 Group ORG + Tracker (other questionnaire data); *n* = 68 Group ORG, *n* = 50 Group ORG + Tracker (BMI); *n* = 85 Group ORG, *n* = 64 Group ORG + Tracker (activity data); *n* = 84 Group ORG, *n* = 62 Group ORG + Tracker (work activity data)

^b^includes one borderline underweight participant

^c^Average weekday work hours were calculated from baseline work diaries

^d^The 36-item version of the Nordic Musculoskeletal Questionnaire [39], modified to measure problems in the last month. Issues identified as causing trouble were collapsed into categories of upper body problems (e.g., neck, shoulders, elbows, and wrists/hands), back problems (e.g., upper back, lower back, and hips/thighs/buttocks) or lower extremity problems (e.g., knees, and ankles/feet)

^e^1 to 10 scale, higher numbers indicate more favourable workplace scores, or greater stress levels

^f^1 to 100 scale, higher numbers indicate better quality of life

**Fig. 1:**
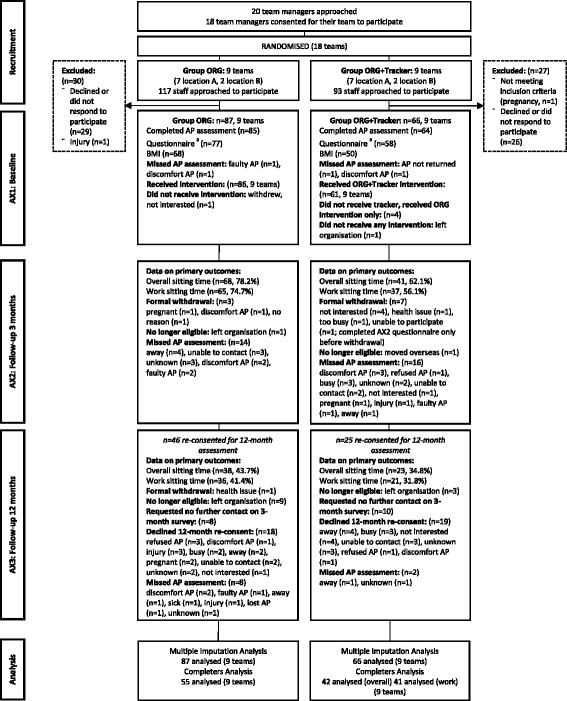
Participant flow chart. AP = activPAL. ^a^ numbers for questionnaires include partial completions. BMI = body mass index, kg/m^2^

The participant flow chart and retention is presented in Fig. 1. By three months, three (2.0 %) participants had become ineligible and 11 (7.2 %) had formally withdrawn. By 12 months there was substantial loss to follow-up: 12 participants (7.8 %) became ineligible by leaving the organisation, 37 (24.2 %) declined the 12-month re-consent, 18 participants (11.8 %) requested no further contact, and one formally withdrew (0.7 %). Additional data loss occurred from participants not completing the activPAL assessments, failing to provide work times in the diaries, or not responding to the online questionnaire. The percentage of participants with any missing activPAL data did not differ significantly (*p* = 0.206) between Group ORG (59.8 %, *n* = 52) and Group ORG + Tracker (69.7 %, *n* = 46). Key reasons for activPAL data loss (out of a total of 126 reasons from 98 participants) included 20 participants being too busy or away during the assessment period/s (22 reasons), 14 participants leaving the organisation, 11 participants developing rash/s from the Hypafix adhesive used to attach the activPAL (12 reasons), 12 participants no longer being interested in the study, and 10 participants being uncontactable, see Fig. 1.

### Intervention fidelity

The emails and information booklet were delivered as intended (see Additional file 9). Further organisational-support components were informally delivered by the champion (e.g., discussions with managers) and intervention fidelity data were not collected. The LUMOback was provided to 61 out of 66 participants with modest uptake (43/61 participants; 70.5 %), and variable self-directed usage in the first 12 weeks (mean ± SD = 12.1 ± 11.6 days; based on 36 participants with valid usage data). LUMOback usage had ceased by 12 months in all study completers. Reasons for non-use included technical difficulties and having no time to set up (see Additional file 9, Part A). The number of participants who participated in promoted strategies (e.g., walking meetings) significantly increased between baseline and three months and between baseline and 12 months (see Additional file 9, Part B).

### Intervention effectiveness and relative effectiveness

#### Three months

For both interventions, the estimated three-month changes (Table 3) in the primary sitting time outcomes were small (<15 min) and not statistically significant. Group ORG did not significantly change in any activity except for overall standing time, which improved by 14.6 min/16 h day (95 % CI: 2.5, 26.8, *p* = 0.018) and Group ORG + Tracker did not significantly change in any activity outcomes over the first three months. The estimated changes were all small (< the MDI) and confidence intervals only encompassed potentially meaningful changes (i.e., including the MDI) for Group ORG + Tracker’s overall standing time and time between sitting bouts, and work hour and overall step counts.

**Table 3:** Three-month mean changes in activity outcomes, and differences between interventions, adjusting for confounders

	Group ORG (*n* = 87)^a^	Group ORG + Tracker (*n* = 66)^a^	Between group difference^a b^(Group ORG + Tracker vs. Group ORG)
Outcome	Adjusted mean change (95 % CI)	Adjusted mean change (95 % CI)	Adjusted mean difference (95 % CI)
Work hours			
Sitting, min/10 h	−3.8 min (−17.4, 9.9), *p* = 0.588	−10.7 min (−26.8, 5.4), *p* = 0.194	−6.6 min (−35.0, 21.7), *p* = 0.645
Prolonged sitting, min/10 h	−9.9 min (−29.8, 9.9), *p* = 0.326	−6.0 min (−33.0, 21.0), *p* = 0.665	+11.1 min (−27.5, 49.8), *p* = 0.572
Time between sitting bouts	+0.2 min (−1.2, 1.7), *p* = 0.761	+0.4 min (−1.3, 2.0), *p* = 0.642	+0.5 min (−2.9, 3.9), *p* = 0.763
Standing, min/10 h	+4.9 min (−6.3, 16.2), *p* = 0.390	+9.4 min (−3.8, 22.5), *p* = 0.162	+3.4 min (−19.8, 26.6), *p* = 0.775
Stepping, min/10 h	−1.1 min (−4.9, 2.8), *p* = 0.580	+1.9 min (−3.4, 7.2), *p* = 0.483	+4.2 min (−6.0, 14.4), *p* = 0.420
Number of steps/10 h	−35.5 steps (−231.8, 160.8), *p* = 0.723	+123.9 steps (−148.6, 396.5), *p* = 0.372	+227.7 steps (−307.8, 763.2), *p* = 0.404
Overall hours			
Sitting, min/16 h	−14.3 min (−29.6, 0.9), *p* = 0.066	−7.4 min (−29.8, 14.9), *p* = 0.513	+1.8 min (−41.9, 45.4), *p* = 0.937
Prolonged sitting, min/16 h	−7.5 min (−26.9, 12.0), *p* = 0.450	−2.8 min (−30.1, 24.5), *p* = 0.841	+6.9 min (−36.0, 49.7), *p* = 0.753
Time between sitting bouts	+0.2 min (−0.3, 0.8), *p* = 0.391	+0.7 min (−0.1, 1.4), *p* = 0.092	+0.9 min (−0.7, 2.4), *p* = 0.266
Standing, min/16 h	**+14.6 min (2.5, 26.8),** ***p*** **= 0.018**	+16.0 min (−1.4, 33.4), *p* = 0.071	+2.1 min (−28.6, 32.8), *p* = 0.892
Stepping, min/16 h	−0.5 min (−6.6, 5.5), *p* = 0.860	−6.5 min (−14.9, 1.9), *p* = 0.131	+1.2 min (−14.4, 16.8), *p* = 0.876
Number of steps/16 h	−42.3 steps (−330.5, 245.8), *p* = 0.773	−259.1 steps (−631.6, 113.3), *p* = 0.172	+60.1 steps (−600.1, 720.4), *p* = 0.858

Significant changes or differences (*p* < 0.05) are in bold

^a^Missing data imputed by chained equations (m = 70 imputations)

^b^Models adjust for baseline values of the outcome and confounders (see Additional file 5)

When comparing their relative effectiveness in the short term, the two interventions showed only small and non-significant differences in activity outcomes, not consistently in favour of one intervention or the other. However, many of the confidence intervals were wide and contained potentially meaningful effects.

#### 12 months

Both interventions resulted in 12-month changes (Table 4) in the primary outcomes that were statistically significant and estimated at approximately half to three-quarters of an hour improvement. Group ORG’s work hour sitting reduction (−40.5 min/10 h, 95 % CI: −60.9, −20.0, *p* < 0.001) appeared to occur primarily through significant increases in standing time, with little evidence of any stepping improvements (+2.4 min/10 h, 95 % CI: −3.0, 7.9, *p* = 0.375). The sitting reduction was also reflected in significantly reduced time accrued in prolonged sitting (−41.3 min/10 h, 95 % CI: −67.9, −14.8, *p* = 0.002) and longer time between sitting bouts at work (+1.7 min/10 h, 95 % CI: 0.3, 3.1, *p* = 0.019). Group ORG’s changes over the entire waking day were largely consistent with their work hour changes. Here sitting changes coincided with significantly improved standing with small non-significant stepping changes, and significant reductions in prolonged sitting time. Confidence intervals around changes for overall time between sitting bouts contained potentially meaningful effects.

**Table 4:** 12-month mean changes in activity outcomes, and differences between interventions, adjusting for confounders

	Group ORG (*n* = 87)^a^	Group ORG + Tracker (*n* = 66)^a^	Between group difference^a b^(Group ORG + Tracker vs. Group ORG)
Outcome	Adjusted mean change (95 % CI)	Adjusted mean change (95 % CI)	Adjusted mean difference (95 % CI)
Work hours			
Sitting, min/10 h	**−40.5 min (-60.9, −20.0),** ***p*** **< 0.001**	**−35.5 min (-60.9, −10.2),** ***p*** **= 0.006**	+4.4 min (−33.1, 41.9), *p* = 0.818
Prolonged sitting, min/10 h	**−41.3 min (−67.9, −14.8),** ***p*** **= 0.002**	**−45.7 min (−84.0, −7.4),** ***p*** **= 0.019**	−1.4 min (−51.2, 48.3), *p* = 0.955
Time between sitting bouts	**+1.7 min (0.3, 3.1),** ***p*** **= 0.019**	+1.5 min (−0.4, 3.3), *p* = 0.114	+0.3 min (−3.0, 3.6), *p* = 0.855
Standing, min/10 h	**+39.2 min (20.9, 57.5),** ***p*** **< 0.001**	**+27.4 min (7.7, 47.1),** ***p*** **= 0.007**	−12.4 min (−44.6, 19.8), *p* = 0.449
Stepping, min/10 h	+2.4 min (−3.0, 7.9), *p* = 0.375	**+9.1 min (0.2, 17.9),** ***p*** **= 0.045**	+7.7 min (−3.8, 19.2), *p* = 0.189
Number of steps/10 h	+123.2 steps (−135.9, 382.4), *p* = 0.350	+433.8 steps (−16.5, 884.1), *p* = 0.059	+369.6 steps (−227.3, 966.4), *p* = 0.224
Overall hours			
Sitting, min/16 h	**−32.1 min (−57.2, −7.0),** ***p*** **= 0.012**	**−35.0 min (−65.6, −4.3),** ***p*** **= 0.025**	−8.4 min (−57.0, 40.3), *p* = 0.735
Prolonged sitting, min/16 h	**−30.0 min (−56.5, −3.6),** ***p*** **= 0.026**	−23.6 min (−54.2, 7.0), *p* = 0.131	+8.0 min (−37.9, 53.9), *p* = 0.732
Time between sitting bouts	+1.1 min (−0.2, 2.4), *p* = 0.097	+1.5 min (−0.4, 3.5), *p* = 0.116	+0.9 min (−1.4, 3.2), *p* = 0.435
Standing, min/16 h	**+33.5 min (13.8, 53.3),** ***p*** **= 0.001**	**+26.9 min (1.1, 52.6),** ***p*** **= 0.041**	−5.1 min (−44.2, 34.0), *p* = 0.797
Stepping, min/16 h	−4.5 min (−13.5, 4.5), *p* = 0.328	+10.0 min (−1.7, 21.7), *p* = 0.095	**+20.6 min (3.1, 38.1),** ***p*** **= 0.021**
Number of steps/16 h	−157.8 steps (−584.3, 268.7), *p* = 0.468	+440.8 steps (−119.3, 1000.9), *p* = 0.123	**+846.5 steps (67.8, 1625.2),** ***p*** **= 0.033**

Significant changes or differences (*p* < 0.05) are in bold

^a^Missing data imputed by chained equations (m = 70 imputations)

^b^Models adjust for baseline values of the outcome and confounders (see Additional file 5)

Group ORG + Tracker’s reductions in sitting time during work hours and across their waking day also coincided with significant increases in standing time, and notably also significantly increased work hour stepping (+9.1 min/10 h, 95 % CI: 0.2, 17.9, *p* = 0.045). There was also a similar but non-significant increase in overall stepping time (+10.0 min/16 h, 95 % CI: −1.7, 21.7, *p* = 0.095). Group ORG + Tracker’s prolonged sitting time reduced significantly during work hours only, with smaller, non-significant changes overall. Confidence intervals around all of Group ORG + Tracker’s non-significant changes included potentially meaningful effects.

When comparing their relative effectiveness in the long term, the ORG + Tracker intervention was significantly more effective than ORG for increasing overall stepping time (+20.6 min/16 h, 95 % CI: 3.1, 38.1, *p* = 0.021) and overall step counts (+846.5 steps/16 h, 95 % CI: 67.8, 1625.2, *p* = 0.033). Other differences were not statistically significant, small, and did not systematically tend to favour either of the interventions. Confidence intervals contained only small effects for work hours sitting; other comparisons were inconclusive.

#### Work and health changes

Neither group showed significant changes in any of the work- or health-related outcomes at three- or 12 months relative to baseline (see Table 5). Estimates of changes were all small for work-related outcomes, with confidence intervals that did not include meaningful changes except for job performance. Estimates of health-related changes were also small, although confidence intervals for Group ORG + Tracker encompassed potentially meaningful effects for stress and physical health quality of life at three months, and stress, physical and mental health quality of life at 12 months.

**Table 5:** Mean three- and 12-month work and health-related changes within Group ORG and Group ORG + Tracker interventions

		Group ORG (*n* = 87)^a^	Group ORG + Tracker (*n* = 66)^a^
Outcome	Time	Adjusted mean change (95 % CI)	Adjusted mean change (95 % CI)
Work outcomes			
Job performance	3 M	−0.1 (−0.4, 0.1), *p* = 0.239	−0.2 (−0.5, 0.1), *p* = 0.318
	12 M	−0.2 (−0.6, 0.3), *p* = 0.476	−0.2 (−0.7, 0.4), *p* = 0.554
Job control	3 M	+0.1 (−0.5, 0.7), *p* = 0.759	−0.2 (−0.9, 0.6), *p* = 0.664
	12 M	−0.1 (−1.6, 1.3), *p* = 0.871	−0.5 (−2.5, 1.6), *p* = 0.650
Work satisfaction	3 M	+0.0 (−0.5, 0.6), *p* = 0.855	+0.1 (−0.5, 0.8), *p* = 0.690
	12 M	−0.3 (−1.4, 0.9), *p* = 0.639	−0.2 (−1.9, 1.4), *p* = 0.788
Health outcomes			
Stress	3 M	−0.1 (−0.8, 0.6), *p* = 0.702	−0.5 (−1.4, 0.4), *p* = 0.309
	12 M	+0.3 (−1.1, 1.6), *p* = 0.705	−0.8 (−2.7, 1.0), *p* = 0.355
Physical health quality of life	3 M	−0.7 (−3.2, 1.8), *p* = 0.604	+1.6 (−1.1, 4.2), *p* = 0.240
	12 M	+2.5 (−4.4, 9.3), *p* = 0.478	−2.7 (−9.7, 4.2), *p* = 0.438
Mental health quality of life	3 M	−0.1 (−3.2, 3.0), *p* = 0.969	−0.6 (−4.1, 3.0), *p* = 0.748
	12 M	−2.1 (−12.8, 8.6), *p* = 0.701	+3.8 (−6.3, 13.9), *p* = 0.463

^a^Missing data imputed by chained equations (m = 80 imputations)

### Sensitivity analyses

Conclusions from the completers analyses (Additional file 6), did not always agree with the main findings. By comparison with the multiple imputation analyses, completers analyses were more likely to show three-month activity changes that were larger and/or statistically significant, which may have reflected a bias from the dropout of healthy participants (Additional file 4). Imputing missing data variably increased and decreased the size and significance of the 12-month activity changes, where there was a high amount of loss to follow-up, produced mostly by not re-consenting into the trial.

### Adverse events

A total of 41 out of 153 participants reported an adverse event, with a total of 45 events reported. The majority of events were reactions to wearing the activity monitor or the activity tracker. At various points in the trial, across the 328 activPAL assessments undertaken, 20 out of 153 participants (13.1 %) reported rashes or reactions to the Hypafix adhesive dressing of different severities: allergic reaction (*n* = 3); rash/very itchy (*n* = 11); and slight/minor irritation or mark (*n* = 6). Of the 43 Group ORG + Tracker participants who wore the LUMOback, 14 (32.6 %) reported the following problems: rash/irritation (*n* = 3); uncomfortable (*n* = 8); or minor back pain or strain from following the posture advice (*n* = 3). Details of the other nine adverse events reported (from eight participants) were reviewed by the chief investigators and all were determined not to have arisen from the study.

## Discussion

This study presents some of the first findings of the impact of organisational support and activity tracker strategies on office workers’ sitting time. An organisational intervention resulted in long-term reductions in sitting time, both overall and specifically during work hours, with or without an activity tracker. This demonstrates that a minimally intensive intervention can be effective for sitting reduction. The organisational intervention primarily replaced sitting time with standing time; the addition of the activity tracker with real-time feedback to the organisational support intervention led to further improvements in stepping, despite the modest and self-directed usage of the activity tracker.

The small, non-significant three-month mean changes in overall sitting and standing (<15 min/16 h day) were consistent with short-duration workplace interventions that implemented educational and behavioural strategies alone [10] or combined with a prompting/self-monitoring tool [45]. The results were less than those found in studies that also utilised sit-stand workstations (>1.5 h/8 h workday sitting reduction, [10]). It may be that for occupations that are primarily desk based, the work environment may limit capacity for change, and office equipment and task design modifications are needed to enable greater changes in sitting and standing during the work day.

One of the key strengths of the study was the examination of the long-term impact of the interventions. Interestingly, the long-term mean changes in sitting, prolonged sitting and standing time of 27–46 min per 10-h workday and 23–35 min per 16-h waking day appeared to be larger than the corresponding short-term changes. Most workplace sitting interventions have typically been of a short duration (e.g., three months) [10] but the few that have examined long-term (8–12 month) changes have found either a reduction or maintenance of effects [46–48] rather than a strengthening. We did not plan *a priori* to compare short and long-term effects, and we had good short term but very low long-term retention. Nonetheless, there are some possible theoretical reasons why stronger effects were observed in the long term compared to the short term that merit discussion. At an organisational level, the intervention strategies may have taken longer than three months to embed sufficiently to generate culture changes. At an individual level, participants may have taken more actions to change their sitting behaviour over time due to factors such as increasing social influence of colleagues and managers who were modelling the intervention strategies [49], and the provision of individualised participant feedback at three months, which may have acted as a ‘wake-up call’. Overall, the success in the long term, within a fairly typical office setting, suggested that behavioural improvement can occur even without environmental modification, however only two locations were examined, and this sustained success may not be true for all workplaces.

Though the intervention included stepping-promotion messages, with the workplace champion including walking strategies in all emails, and providing a step count guide, the organisational intervention alone did not result in a significant or meaningful improvement in stepping time either in the short or long term. This finding was consistent with previous studies that used the *Stand Up Australia* intervention from which the current intervention was based [16, 17, 46]. Notably, the addition of an activity tracker — one selected for its real-time sitting/upright posture feedback — significantly enhanced the organisational intervention’s impact on stepping time and step counts. The improvement within the group receiving a tracker and the organisational intervention was approximately 10 min per day, or just over an hour per week. This finding is consistent with other workplace studies that have used pedometers or other activity trackers in addition to organisational-support strategies [24, 50, 51]. Activity trackers may be an effective addition to physical activity and/or sedentary reduction interventions in office workers; feasible, acceptable, effective, and cost-effective options should be identified and evaluated.

Though neither intervention was significantly more effective on the non-stepping outcomes, there are several considerations that may indicate that it is premature to consider that the LUMOback or other such trackers are unlikely to enhance an organisational intervention’s effectiveness for outcomes other than stepping. Firstly, the study did not power *a priori* on relative effectiveness and several comparisons were inconclusive. Secondly, although we randomised teams, we do not know enough about the way in which the teams were co-located or interacted to determine that the usage of the tracker by Group ORG + Tracker did not impact on behaviour of Group ORG (for example, a Group ORG + Tracker participant transitions to a standing position on account of the tracker’s prompting, which is noticed by a co-worker from Group ORG, who also stands). Finally, the low uptake of the LUMOback coupled with self-directed usage, and the discontinuation of use by 12 months may have limited its effectiveness. Discontinuation consistently occurs across multiple settings and trackers [52, 53], but our trial also had lower uptake of the tracker than other activity tracker trials [23, 24]. The same tracker used or promoted in a different manner, the use of a different tracker, and/or the use of trackers selected and/or purchased by the participant rather than the organisation [53] may have a different impact on behaviour, uptake and usage than we observed. Providing in-person set up support may also improve uptake and possibly effectiveness, since technical difficulties and set up time were some of the contributors to low uptake.

Neither intervention resulted in significant changes in work or health outcomes. This finding is consistent with reviews that have found no negative impact of workplace sitting interventions on work performance [54–56] or health [56] and is appropriate given the minimal nature of the interventions. However, changes in job performance, stress, and quality of life were inconclusive so more studies, with adequate power, are needed to corroborate these findings.

### Study strengths and limitations

A key strength of this intervention was its pragmatic and context-specific implementation, where change was primarily driven by the organisation rather than the researchers. As such, the findings are likely to be reflective of change that can be achieved in similar interventions. Although this study was conducted in a large organisation, the minimal intervention was designed to be tailored and adaptable, including for use in smaller businesses, where resources for health promotion may be scarce [57].

Another key strength of the study was the use of a best-practice objective measure for the primary outcomes, and other activity outcomes. Furthermore, the time most potentially impacted by the intervention (time at work) was considered in addition to overall waking hours. Many workplace trials neglect to evaluate both activity during work hours and across the whole day. This is despite the fact that activity changes may need to be evident overall to improve health, and these may be much smaller than effects at work due to work constituting only a fraction of our total time and possible compensation (i.e., participants moving less outside of work hours to compensate for extra activity at work) [58].

This study also had limitations. Firstly, the evaluation of the effectiveness of each intervention is limited to a pre-post design as there was no control group. Changes could represent natural changes over time that would have occurred in absence of intervention or other biases, not effectiveness. The high 12-month data loss was similar to levels reported within other workplace lifestyle intervention trials [15, 59, 60]. Biases from missing data (which were evident in the difference between imputation and completers results) were minimised by the use of multiple imputation analyses, however, the imputed estimates cannot be considered completely unbiased, and the multiple imputation methodology is not without limitations, such as outlined by White et al. [42]. Although many potential confounders were evaluated, residual confounding is still possible from unmeasured data. The interventions were delivered in only one organisation which limits generalisability. The organisation also has a key focus on employee health and was likely already receptive to workplace health improvements. Additional initial strategies focusing on knowledge and cultural change may be needed in workplaces without a champion or health focus. There is also a possibility for selection bias as participants were not a random sample of employees. Team managers were approached to participate and employees self-selected themselves into the intervention, leading to the possibility of more motivated participants than the broader Lendlease population. Characteristics of employees who were not approached or who declined the interventions were not collected, so selection bias cannot be formally evaluated. However, participant characteristics were diverse and baseline work activity levels were similar to office workers in other studies [16, 17, 46], indicating that any bias may have been minimal. Acceptability and feasibility of the interventions, self-directed usage patterns of the LUMOback, and specific participant and intervention characteristics that led to behaviour change are yet to be evaluated and warrant further attention.

## Conclusions

In conclusion, we have demonstrated that worksite-driven and internally delivered organisational-support strategies are an effective sitting-reduction strategy in the long term (12 months). That the strategies were delivered internally by the workplace champion bodes well for their sustainability. The minimally intensive nature of these strategies also enables them to be implemented and disseminated in other workplaces. Activity trackers may also play a role in increasing activity in the workplace.

## Additional files

Additional file 1: CONSORT Extension for Cluster Trials and TIDieR Checklists. (DOCX 47 kb)
Additional file 2: ﻿Intervention email. (PDF 255 kb)
Additional file 3: Assessment and intervention dates. (DOCX 12 kb)
Additional file 4: Predictors of missing data. (DOCX 23 kb)
Additional file 5: Confounders adjusted for in analyses. (DOCX 15 kb)
Additional file 6: Mean adjusted within group changes at three and 12 months from completers analyses. (DOCX 15 kb)
Additional file 7: Unadjusted within group changes, and differences between interventions, at three and 12 months. (DOCX 17 kb)
Additional file 8: Part A: Uptake, baseline characteristics and activPAL completion by team. ​Part B: ICCs (95 % CI) for team clustering at baseline. (DOCX 27 kb)
Additional file 9: Part A. Delivery and use of intervention components. Part B. Any use of promoted strategies. Part C. Any use of other strategies. (DOCX 18 kb)

## Abbreviations

AP: activPAL
app: Mobile application
BMI: Body mass index
CEO: Chief executive officer
FMI: Fraction of missing information
FTE: Full-time equivalent
ICC: Intra-cluster correlation coefficient
MDI: Mean difference of interest
SF: Short form
