# Supplementary material for: Evaluating the effectiveness of organisational-level strategies with or without an activity tracker to reduce office workers’ sitting time: a cluster-randomised trial
Source: Int J Behav Nutr Phys Act. 2016 Nov 4;13:115. doi: 10.1186/s12966-016-0441-3 (PMC5097432; doi:10.1186/s12966-016-0441-3)
Supplement: Additional file 2: — ﻿Intervention email. (PDF 255 kb) [file 12966_2016_441_MOESM2_ESM.pdf]

STAND UP – SIT LESS – MOVE MORE

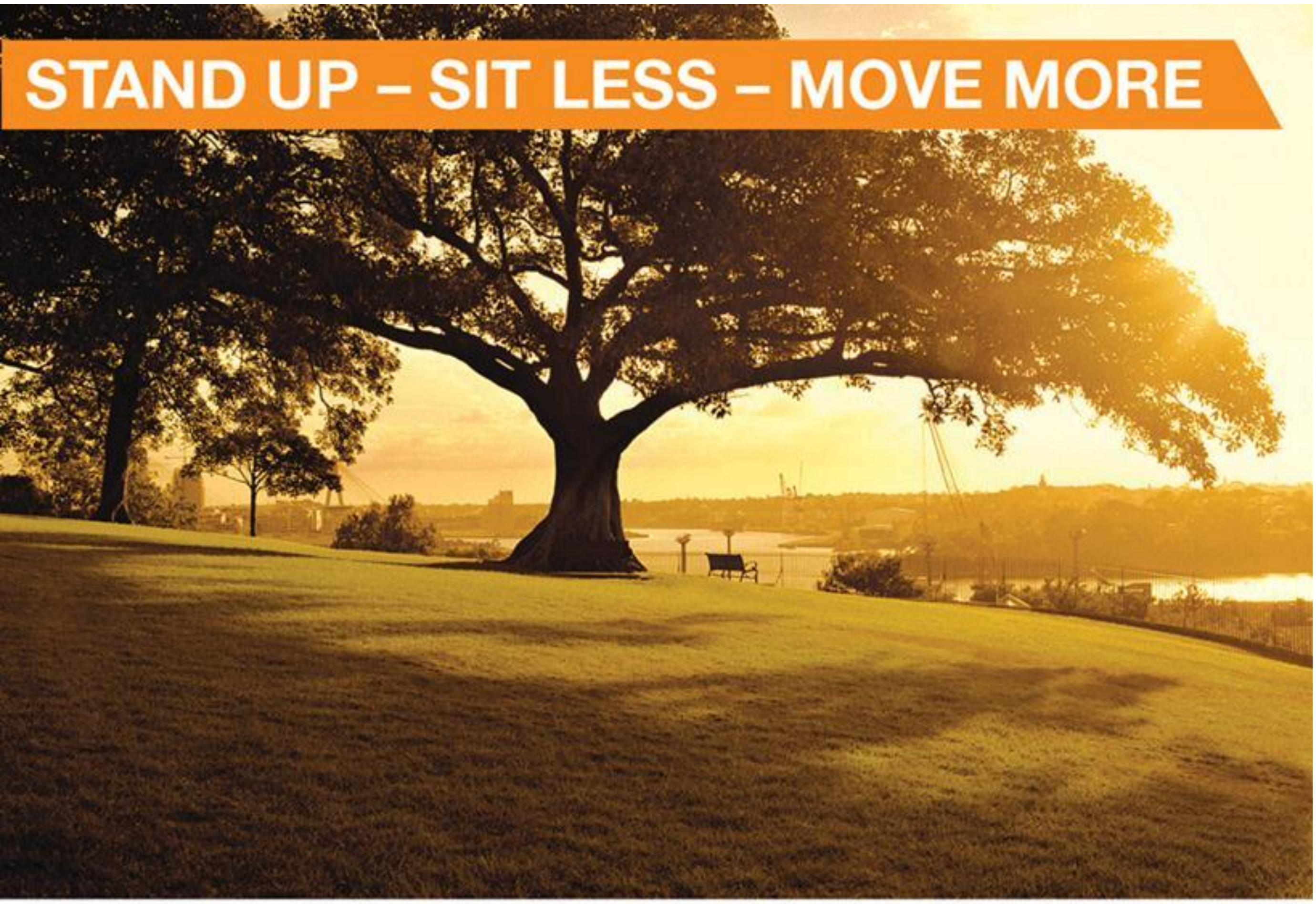

Welcome to week six of the 'Stand Up Lend Lease' trial which aims to increase physical activity during your day.

One senior manager I spoke with last week said he was now holding regular walking meetings. "I am now having 2-3 walking meetings per week. Perfect for the 1:1 personal development conversations that we hold fortnightly. Everybody loves the concept and I've never seen so much of Sydney harbour.

It beats sucking down coffee in the café or yet another hour in a meeting room.

Did you know the average steps taken by Lend Lease people in baseline period was 5,065 steps per day.

HOW MANY STEPS ARE ENOUGH?

Steps Per Day	Activity Level
<5,000	sedentary
5,000 - 7,499	low active
7,500 - 9,999	somewhat active
>10,000	active
>12,500	highly active

1 Tudor-Locke, C., & Bassett, D.R. Jr. (2004). How Many Steps/Day Are Enough? Preliminary Pedometer Indices for Public Health. Sports Medicine, 34(1): 1-8.

TIP: Have a walking meeting to increase your step count.

Baker IDI HEART & DIABETES INSTITUTE

THE UNIVERSITY OF QUEENSLAND AUSTRALIA

Lend Lease
