# Supplementary material for: Evaluating the effectiveness of organisational-level strategies with or without an activity tracker to reduce office workers’ sitting time: a cluster-randomised trial
Source: Int J Behav Nutr Phys Act. 2016 Nov 4;13:115. doi: 10.1186/s12966-016-0441-3 (PMC5097432; doi:10.1186/s12966-016-0441-3)
Supplement: Additional file 3: — Assessment and intervention dates. (DOCX 12 kb) [file 12966_2016_441_MOESM3_ESM.docx]

| Assessment Point | Months since intervention | Dates |
| --- | --- | --- |
| Location A |  |  |
| AX1 (Baseline) | -1 | March-April 2014 |
| Intervention begins | 0 | April 2014 |
| AX2 monitor assessment | 3-5 | July-September 2014 |
| AX2 questionnaire | 5-8 | September-December 2014 |
| AX3 monitor assessment | 13-16 | May-August 2015 |
| AX3 questionnaire | 13-16 | May-August 2015 |
| Location B |  |  |
| AX1 (Baseline) | -1 | April 2014 |
| Intervention begins | 0 | May 2014 |
| AX2 monitor assessment | 4-7 | September-December 2014 |
| AX2 questionnaire | 4-7 | September-December 2014 |
| AX3 monitor assessment | 12-14 | May-July 2015 |
| AX3 questionnaire | 12-15 | May-August 2015 |

Additional file 3. Assessment and intervention dates
