# Supplementary material for: Evaluating the effectiveness of organisational-level strategies with or without an activity tracker to reduce office workers’ sitting time: a cluster-randomised trial
Source: Int J Behav Nutr Phys Act. 2016 Nov 4;13:115. doi: 10.1186/s12966-016-0441-3 (PMC5097432; doi:10.1186/s12966-016-0441-3)
Supplement: Additional file 4: — Predictors of missing data. (DOCX 23 kb) [file 12966_2016_441_MOESM4_ESM.docx]

Additional file 4: Logistic regression analyses for baseline variables predicting missing data for models of activPAL-assessed activity (Part A) and the questionnaire-assessed work and health outcomes (Part B).

Part A

| Variables | Overall or by Group ^a^ | Three-month activity outcomes ^b^ | | Overall or by Group ^a^ | 12-month activity outcomes ^b^ | |
| --- | --- | --- | --- | --- | --- | --- |
|  |  | *B* | *SE* |  | *B* | *SE B* |
| Age | Overall | -.007 | .021 | Overall | -.017 | .022 |
| Female sex | Overall | -.390 | .326 | Overall | **.492#** | **.356** |
| University education | Overall | -.203 | .455 | Overall | **-.999#** | **.582** |
| Manager | Overall | -.043 | .561 | Overall | -.215 | .587 |
| Weekday work hours/day ^c^ | Overall | -.049 | .147 | Overall | -.022 | .156 |
| *Job category* |  |  |  |  |  |  |
| Senior leader vs. manager | Overall | -.254 | .519 | Overall | -.401 | .537 |
| Other vs. manager | Overall | -.591 | .364 | Overall | -.064 | .390 |
|  |  |  |  |  |  |  |
| *Workplace variables* |  |  |  |  |  |  |
| Job performance ^d^ | Overall | **.253#** | **.197** | Overall | **.522*** | **.210** |
| Job control ^d^ | Overall | **.158#** | **.103** | Overall | -.086 | .104 |
| Supervisor relations ^de^ | Overall | -.001 | .099 | Overall | .059 | .102 |
| Work satisfaction ^d^ | Overall | .086 | .119 | Overall | .061 | .123 |
|  |  |  |  |  |  |  |
| *Psychosocial variables* |  |  |  |  |  |  |
| Sitting knowledge ^f^ | Overall | **-.859*** | **.347** | Overall | **-.745*** | **.358** |
| Preference for sitting at work, 50% or more of time ^f^ | Overall | .260 | .371 | Overall | .043 | .387 |
|  |  |  |  |  |  |  |
| *Strategy use at baseline* |  |  |  |  |  |  |
| App/device use | Overall | .398 | .390 | Overall | .166 | .412 |
| Use of workplace strategies ^g^ | Group ORG | **-1.117#** | **.722** | Overall | **-.677#** | **.496** |
|  | Group ORG+Track | **.984#** | **.715** |  |  |  |
|  |  |  |  |  |  |  |
| *Health* |  |  |  |  |  |  |
| Smoking | Overall | .767 | .599 | Overall | .667 | .685 |
| Stress ^d^ | Overall | .039 | .078 | Overall | -.046 | .082 |
| Back problems ^h^ | Overall | **-.834*** | **.355** | Overall | -.262 | .365 |
| Lower extremity problems ^h^ | Overall | **-.637#** | **.381** | Group ORG | .070 | .483 |
|  |  |  |  | Group ORG+Track | **-1.327*** | **.631** |
| Upper body problems ^h^ | Group ORG | **-1.339**** | **.498** | Overall | **-.539#** | **.385** |
|  | Group ORG+Track | -.342 | .550 |  |  |  |
|  |  |  |  |  |  |  |
| Mental health quality of life | Group ORG | **.035#** | **.023** | Overall | **.032#** | **.016** |
|  | Group ORG+Track | -.030 | .027 |  |  |  |
|  |  |  |  |  |  |  |
| Physical health quality of life | Group ORG | **-.054#** | **.031** | Group ORG | **-.066#** | **.037** |
|  | Group ORG+Track | .038 | .041 | Group ORG+Track | .024 | .042 |
|  |  |  |  |  |  |  |
|  |  |  |  |  |  |  |
| BMI (kg/m^2^) | Group ORG | **.172*** | **.082** | Overall | .008 | .055 |
|  | Group ORG+Track | **-.165#** | **.101** |  |  |  |
|  |  |  |  |  |  |  |
| *Activity outcomes* |  |  |  |  |  |  |
| Overall sitting | Group ORG | .004 | .003 | Group ORG | .002 | .003 |
|  | Group ORG+Track | **-.008#** | **.004** | Group ORG+Track | **-.007#** | **.005** |
|  |  |  |  |  |  |  |
| Overall standing | Group ORG | -.003 | .004 | Overall | .000 | .003 |
|  | Group ORG+Track | **.007#** | **.005** |  |  |  |
|  |  |  |  |  |  |  |
| Overall stepping | Group ORG | -.009 | .008 | Group ORG | -.002 | .008 |
|  | Group ORG+Track | **.015#** | **.010** | Group ORG+Track | **.018#** | **.012** |

Part B

| Variables | Overall or by Group ^a^ | Three-month work/health  outcomes ^b^ | | Overall or by  Group ^a^ | 12-month work/health outcomes ^b^ | |
| --- | --- | --- | --- | --- | --- | --- |
|  |  | *B* | *SE B* |  | *B* | *SE B* |
| Age | Overall | -.004 | .021 | Overall | .004 | .023 |
| Female sex | Overall | **-.467#** | **.327** | Overall | .075 | .357 |
| University education | Overall | .260 | .465 | Overall | **-.890#** | **.583** |
| Manager | Overall | -.216 | .566 | Overall | -.318 | .588 |
| Weekday work hours/day ^c^ | Overall | -.068 | .148 | Overall | -.130 | .158 |
| *Job category* |  |  |  |  |  |  |
| Senior leader vs. manager | Overall | -.338 | .521 | Group ORG | **-1.253#** | **.644** |
|  |  |  |  | Group ORG+Track ^i^ |  |  |
| Other vs. manager | Overall | -.538 | .365 | Group ORG | **-.965#** | **.524** |
|  |  |  |  | Group ORG+Track | **.932#** | **.710** |
|  |  |  |  |  |  |  |
| *Workplace variables* |  |  |  |  |  |  |
| Job performance ^d^ | Group ORG | -.034 | .259 | Overall | **.414*** | **.207** |
|  | Group ORG+Track | **.531#** | **.345** |  |  |  |
| Job control ^d^ | Overall | **.155#** | **.105** | Overall | .065 | .103 |
| Supervisor relations ^de^ | Overall | .074 | .103 | Overall | .020 | .104 |
| Work satisfaction ^d^ | Overall | -.037 | .120 | Overall | -.079 | .126 |
|  |  |  |  |  |  |  |
| *Psychosocial variables* |  |  |  |  |  |  |
| Sitting knowledge ^f^ | Overall | **-.733*** | **.343** | Overall | **-.897*** | **.369** |
| Preference for sitting at work, 50% or more of time ^f^ | Overall | .068 | .375 | Overall | .051 | .393 |
|  |  |  |  |  |  |  |
| *Strategy use at baseline* |  |  |  |  |  |  |
| App/device use | Overall | .049 | .396 | Overall | -.424 | .402 |
| Use of workplace strategies ^g^ | Group ORG | **-1.107#** | **.727** | Overall | **-.693#** | **.501** |
|  | Group ORG+Track | .191 | .682 |  |  |  |
|  |  |  |  |  |  |  |
| *Health* |  |  |  |  |  |  |
| Smoking | Overall | **.937#** | **.600** | Overall | .130 | .631 |
| Stress ^d^ | Overall | -.011 | .079 | Overall | -.102 | .084 |
| Back problems ^h^ | Overall | **-.494#** | **.354** | Overall | -.365 | .373 |
| Lower extremity problems ^h^ | Overall | -.272 | .378 | Overall | **-.495#** | **.383** |
| Upper body problems ^h^ | Overall | -.214 | .362 | Group ORG | **-.978#** | **.522** |
|  |  |  |  | Group ORG+Track | .077 | .609 |
|  |  |  |  |  |  |  |
| Mental health quality of life | Overall | .004 | .016 | Overall | **.047**** | **.017** |
|  |  |  |  |  |  |  |
|  |  |  |  |  |  |  |
| Physical health quality of life | Overall | -.017 | .024 | Group ORG | **-.095*** | **.042** |
|  |  |  |  | Group ORG+Track | -.001 | .044 |
|  |  |  |  |  |  |  |
|  |  |  |  |  |  |  |
| BMI (kg/m^2^) | Group ORG | **.108#** | **.078** | Group ORG | **.113#** | **.076** |
|  | Group ORG+Track | -.062 | .094 | Group ORG+Track | -.065 | .088 |
|  |  |  |  |  |  |  |
| *Activity outcomes* |  |  |  |  |  |  |
| Overall sitting | Group ORG | .003 | .003 | Overall | .000 | .003 |
|  | Group ORG+Track | **-.005#** | **.004** |  |  |  |
|  |  |  |  |  |  |  |
| Overall standing | Group ORG | -.002 | .004 | Overall | -.001 | .003 |
|  | Group ORG+Track | **.007#** | **.005** |  |  |  |
|  |  |  |  |  |  |  |
| Overall stepping | Overall | -.006 | .006 | Group ORG | -.005 | .008 |
|  |  |  |  | Group ORG+Track | **.016#** | **.012** |

Table presents association of predictors with missing data from logistic regression models. All predictors of missing data significant at p<0.2 (highlighted in bold) were subsequently used to predict missing outcome values via multiple imputation except when this was not possible due to: collinearity (e.g., models of activity do not adjust for other activities); or non-convergence or other problems with the imputation models (i.e., job control omitted from overall prolonged sitting model, back problems omitted from work prolonged sitting model).

#p<0.2 *p<0.05 **p<0.01

^a^ Effects are reported separately by group when the interaction (predictor x group) reached p<0.2 and the predictor was significant at p<0.2 within one or both groups.

^b^ Models required outcome data at baseline, the relevant follow-up, and covariates.

^c^ Average weekday work hours were calculated from baseline work diaries

^d^ 1 to 10 scale, higher numbers indicate more favourable workplace scores, or greater stress levels

^e^ derived from an average of two items from the Health and Work Questionnaire [36]

^f^ Stand Up Australia measures [28]; sitting knowledge = knowledge of the health impacts of sitting, 5 point scale, 1 = worst to 5 = best.

^g^ Frequency of use of workplace strategies to reduce sitting and increase activity (mean of all strategies, 5 point scale, 1 = never to 5 = very often).

^h^ The 36-item version of the Nordic Musculoskeletal Questionnaire [38], modified to measure problems in the last month, was used to measure musculoskeletal health. Issues identified as causing trouble were collapsed into categories of upper body problems (e.g., neck, shoulders, elbows, and wrists/hands), back problems (e.g., upper back, lower back, and hips/thighs/buttocks) or lower extremity problems (e.g., knees, and ankles/feet).

^i^ Data were missing from all senior leaders in Group ORG+Track at 12 months, cell size = 0.
