# Supplementary material for: Evaluating the effectiveness of organisational-level strategies with or without an activity tracker to reduce office workers’ sitting time: a cluster-randomised trial
Source: Int J Behav Nutr Phys Act. 2016 Nov 4;13:115. doi: 10.1186/s12966-016-0441-3 (PMC5097432; doi:10.1186/s12966-016-0441-3)
Supplement: Additional file 5: — Confounders adjusted for in analyses. (DOCX 15 kb) [file 12966_2016_441_MOESM5_ESM.docx]

Additional file 5: Confounders adjusted for in analyses

| Outcome | Models adjusted for ^a^ |
| --- | --- |
| *Work hours* |  |
| Work sitting, min/10h workday | Baseline work sitting, age, sex (female/male), physical QoL score (low/high)^b^, job performance score, work satisfaction score, back problems (yes/no)^c^ |
| Work prolonged sitting, min/10h workday | Baseline work prolonged sitting, age, sex (female/male), job control score, job performance score, currently smoke (yes/no) |
| Time between sitting bouts | Baseline time between sitting bouts, age, sex (female/male), physical QoL score (low/high), job performance score, supervisor relations score ^d^, work satisfaction score, BMI, back problems (yes/no) |
| Work standing, min/10h workday | Baseline work standing, age, sex (female/male), physical QoL score (low/high), job performance score, work satisfaction score, back problems (yes/no) |
| Work stepping, min/10h workday | Baseline work stepping, age, sex (female/male), physical QoL score (low/high), mental QoL score (low/mid/high)^e^, job performance score, work satisfaction score, stress, BMI, job category (senior leader/manager/other), completed university education (yes/no), upper body problems (yes/no) |
| Number of steps/10h workday | Baseline work step count, age, sex (female/male), physical QoL score (low/high), mental QoL score (low/mid/high), job performance score, work satisfaction score, stress, BMI, job category (senior leader/manager/other), completed university education (yes/no), upper body problems (yes/no) |
| *Overall hours* |  |
| Overall sitting, min/16h day | Baseline sitting, age, sex (female/male), physical QoL score (low/high), work satisfaction score, completed university education (yes/no), back problems (yes/no), weekday work hours/day^f^ |
| Overall prolonged sitting, min/16h day | Baseline prolonged sitting, age, sex (female/male), physical QoL score (low/high), job performance score, work satisfaction score |
| Time between sitting bouts | Baseline time between sitting bouts, age, sex (female/male), physical QoL score (low/high), mental QoL score (low/mid/high), work satisfaction score, supervisor relations score, BMI, currently smoke (yes/no), back problems (yes/no), lower extremity problems (yes/no) |
| Overall standing, min/16h day | Baseline standing, age, sex (female/male), physical QoL score (low/high), job performance score, work satisfaction score, supervisor relations score, weekday work hours/day |
| Overall stepping, min/16h day | Baseline stepping, age, sex (female/male), physical QoL score (low/high), mental QoL score (low/mid/high), job control score, work satisfaction score, stress, currently smoke (yes/no), job category (senior leader/manager/other), completed university education (yes/no), upper body problems (yes/no), lower extremity problems (yes/no) |
| Number of steps/16h day | Baseline step count, age, sex (female/male), physical QoL score (low/high), mental QoL score (low/mid/high), job control score, work satisfaction score, stress, currently smoke (yes/no), job category (senior leader/manager/other), completed university education (yes/no), upper body problems (yes/no), lower extremity problems (yes/no) |
| *Work outcomes* |  |
| Job performance | Baseline job performance, age, sex (female/male), stress, physical QoL score (low/high), mental QoL score (low/mid/high), BMI, completed university education (yes/no), job category (senior leader/manager/other) |
| Job control | Baseline job control, age, sex (female/male), job performance score, supervisor relations score, total sitting time, BMI, currently smoke (yes/no), job category (senior leader/manager/other) |
| Work satisfaction | Baseline work satisfaction, age, sex (female/male), mental QoL score (low/mid/high), job performance score, completed university education (yes/no), back problems (yes/no), lower extremity problems (yes/no) |
| *Health outcomes* |  |
| Stress | Baseline stress, age, sex (female/male), job performance score, job category (senior leader/manager/other), BMI, back problems (yes/no) |
| Physical health QoL | Baseline physical QoL, age, sex (female/male), mental QoL score (low/mid/high), job performance score |
| Mental health QoL | Baseline mental QoL, age, sex (female/male), physical QoL score (low/mid/high), back problems (yes/no) |

^a^ Between-group differences were estimated from models that adjust for baseline values, age (years), sex (female/male) and other potential confounders if they were significant at p<0.2 (backward elimination): physical health quality of life (QoL) score (1-100); mental health quality of life (QoL) score (1-100); job control score (1-10); job performance score (1-10); supervisor relations score (1-10); work satisfaction score (1-10); stress (1-10); body mass index (BMI: kg/m^2^); completed university education (yes/no); currently smoke (yes/no); back problems (yes/no); upper body problems (yes/no); lower extremity problems (yes/no); job category (senior leader/manager/other). Activity outcomes during all hours, and work- and health outcomes were also tested for average baseline weekday work hours/day. Work and health outcomes were also tested for baseline total sitting time. Team was included as a random intercept to correct for clustering.

^a^ Physical health quality of life was categorised into two categories, 0 to <50/50 to 100.

^c^ The 36-item version of the Nordic Musculoskeletal Questionnaire [38], modified to measure problems in the last month, was used to measure musculoskeletal health. Issues identified as causing trouble were collapsed into categories of upper body problems (e.g., neck, shoulders, elbows, and wrists/hands), back problems (e.g., upper back, lower back, and hips/thighs/buttocks) or lower extremity problems (e.g., knees, and ankles/feet).

^d^ derived from an average of two items from the Health and Work Questionnaire [36]

^e^ Mental health quality of life was categorised into three categories, 0 to <35/35 to <50/50 to 100.

^f^ Average weekday work hours/day were calculated from baseline work diaries.
