# Supplementary material for: Evaluating the effectiveness of organisational-level strategies with or without an activity tracker to reduce office workers’ sitting time: a cluster-randomised trial
Source: Int J Behav Nutr Phys Act. 2016 Nov 4;13:115. doi: 10.1186/s12966-016-0441-3 (PMC5097432; doi:10.1186/s12966-016-0441-3)
Supplement: Additional file 6: — Mean adjusted within group changes at three and 12 months from completers analyses. (DOCX 15 kb) [file 12966_2016_441_MOESM6_ESM.docx]

Additional file 6. Mean adjusted within group changes at three and 12 months from completers analyses.

|  |  | Group ORG | Group ORG+Tracker |
| --- | --- | --- | --- |
| Outcome | Time | Adjusted mean change (95%CI) | Adjusted mean change (95%CI) |
| **Work hours** |  | n=55 | n=41 |
| Sitting, min/10h | 3M | -4.3 min (-20.1, 11.5), p=0.590 | **-16.1 min (-31.9, -0.3), p=0.045** |
| Prolonged sitting, min/10h | 3M | -9.5 min (-33.5, 14.4), p=0.435 | -12.7 min (-42.9, 17.5), p=0.409 |
| Time between sitting bouts | 3M | -0.3 min (-1.0, 0.4), p=0.414 | **+0.5 min (0.0, 1.0), p=0.040** |
| Standing, min/10h | 3M | +5.5 min (-7.6, 18.5), p=0.411 | +10.6 min (-2.5, 23.6), p=0.114 |
| Stepping, min/10h | 3M | -1.2 min (-6.0, 3.5), p=0.615 | **+6.4 min (1.0, 11.7), p=0.019** |
| Number of steps/10h | 3M | -41.4 steps (-268.5, 185.7), p=0.721 | **+381.6 steps (107.7, 655.5), p=0.006** |
| **Overall hours** |  | n=55 | n=42 |
| Sitting, min/16h | 3M | -11.9 min (-30.7, 6.8), p=0.211 | **-28.3 min (-55.2, -1.5), p=0.039** |
| Prolonged sitting, min/16h | 3M | -8.4 min (-33.4, 16.6), p=0.510 | -10.8 min (-39.9, 18.4), p=0.470 |
| Time between sitting bouts | 3M | +0.1 min (-0.6, 0.8), p=0.814 | **+0.9 min (0.3, 1.5), p=0.002** |
| Standing, min/16h | 3M | **+14.8 min (0.1, 29.5), p=0.049** | **+27.4 min (6.1, 48.7), p=0.012** |
| Stepping, min/16h | 3M | -2.7 min (-10.3, 4.8), p=0.479 | -1.1 min (-11.3, 9.0), p=0.826 |
| Number of steps/16h | 3M | -145 steps (-505, 215), p=0.430 | -21.5 steps (-467.3, 424.4), p=0.925 |
| **Work hours** |  | n=55 | n=41 |
| Sitting, min/10h | 12M | **-44.1 min (-69.9, -18.3), p=0.001** | **-27.2 min (-50.7, -3.6), p=0.024** |
| Prolonged sitting, min/10h | 12M | **-45.5 min (-72.4, -18.7), p=0.001** | **-64.3 min (-110.7, -17.8), p=0.007** |
| Time between sitting bouts | 12M | **+1.6 min (0.2, 3.0), p=0.021** | **+1.1 min (0.2, 2.0), p=0.019** |
| Standing, min/10h | 12M | **+42.7 min (19.5, 65.9), p<0.001** | **+23.5 min (4.8, 42.2), p=0.014** |
| Stepping, min/10h | 12M | +2.7 min (-3.6, 9.0), p=0.402 | +5.9 min (-1.6, 13.4), p=0.124 |
| Number of steps/10h | 12M | +85.3 steps (-193.7, 364.4), p=0.549 | +321.4 steps (-74.3, 717.1), p=0.111 |
| **Overall hours** |  | n=55 | n=42 |
| Sitting, min/16h | 12M | -24.7 min (-50.8, 1.4), p=0.063 | -5.8 min (-30.1, 18.6), p=0.643 |
| Prolonged sitting, min/16h | 12M | -24.3 min (-52.5, 3.9), p=0.092 | -16.2 min (-48.9, 16.5), p=0.333 |
| Time between sitting bouts | 12M | +0.8 min (-0.3, 1.9), p=0.136 | **+0.8 min (0.0, 1.5), p=0.045** |
| Standing, min/16h | 12M | **+29.3 min (6.8, 51.8), p=0.011** | +12.9 min (-9.5, 35.2), p=0.258 |
| Stepping, min/16h | 12M | -6.1 min (-15.1, 2.9), p=0.185 | -4.0 min (-11.9, 3.9), p=0.325 |
| Number of steps/16h | 12M | -335.6 steps (-753.5, 82.3), p=0.115 | -189.2 steps (-616.5, 238.1), p=0.385 |

Significant changes (p<0.05) are in bold.

Adjusted for predictors of missing data, see Additional File 4.
