# Supplementary material for: Evaluating the effectiveness of organisational-level strategies with or without an activity tracker to reduce office workers’ sitting time: a cluster-randomised trial
Source: Int J Behav Nutr Phys Act. 2016 Nov 4;13:115. doi: 10.1186/s12966-016-0441-3 (PMC5097432; doi:10.1186/s12966-016-0441-3)
Supplement: Additional file 7: — Unadjusted within group changes, and differences between interventions, at three and 12 months. (DOCX 17 kb) [file 12966_2016_441_MOESM7_ESM.docx]

Additional file 7. Unadjusted within group changes, and differences between interventions, at three and 12 months.

|  |  | Group ORG | Group ORG+Tracker | Between group difference (Group ORG+Tracker vs. Group ORG) |
| --- | --- | --- | --- | --- |
| Outcome | Time | Mean change (95%CI) | Mean change (95%CI) | Mean difference (95%CI) |
| **Work hours** |  |  |  |  |
| Sitting, min/10h | 3M | -4.6 min (-21.8, 12.5), p=0.597 | -13.2 min (-34.3, 7.9), p=0.220 | -8.6 min (-35.7, 18.5), p=0.535 |
| Prolonged sitting, min/10h | 3M | -10.8 min (-34.7, 13.0), p=0.372 | -6.4 min (-35.3, 13.0), p=0.372 | +4.4 min (-32.9, 41.8), p=0.817 |
| Time between sitting bouts | 3M | +0.3 min (-1.5, 2.2), p=0.738 | +0.6 min (-1.6, 2.8), p=0.598 | +0.3 min (-2.7, 3.2), p=0.853 |
| Standing, min/10h | 3M | +5.3 min (-7.8, 18.3), p=0.212 | +10.6 min (-6.0, 27.1), p=0.429 | +5.3 min (-15.6, 26.1), p=0.619 |
| Stepping, min/10h | 3M | -0.9 min (-6.8, 4.9), p=0.751 | +2.6 min (-4.4, 9.6), p=0.464 | +3.5 min (-5.6, 12.7), p=0.448 |
| Number of steps/10h | 3M | -31.0 steps (-321.9, 259.9), p=0.835 | +158.3 steps (-194.7, 511.3), p=0.379 | +189.3 steps (-272.7, 651.3), p=0.422 |
| **Overall hours** |  |  |  |  |
| Sitting, min/16h | 3M | -15.5 min (-41.3, 10.3), p=0.240 | -13.6 min (-43.3, 16.1), p=0.368 | +1.8 min (-37.2, 40.9), p=0.926 |
| Prolonged sitting, min/16h | 3M | -8.4 min (-36.3, 19.4), p=0.553 | -4.1 min (-37.7, 29.5), p=0.809 | +4.3 min (-39.6, 48.2), p=0.847 |
| Time between sitting bouts | 3M | +0.2 min (-0.6, 1.0), p=0.576 | +0.8 min (-0.2, 1.8), p=0.105 | +0.6 min (-0.7, 1.9), p=0.351 |
| Standing, min/16h | 3M | +15.0 min (-3.0, 33.0), p=0.102 | +19.5 min (-1.9, 41.0), p=0.074 | +4.5 min (-22.9, 31.9), p=0.747 |
| Stepping, min/16h | 3M | +0.0 min (-9.9, 10.0), p=0.994 | -5.3 min (-16.8, 6.3), p=0.373 | -5.3 min (-20.4, 9.9), p=0.493 |
| Number of steps/16h | 3M | -23.2 steps (-474.3, 428.0), p=0.920 | -220.2 steps (-734.1, 293.7), p=0.401 | -197.0 steps (-882.9, 488.9), p=0.573 |
| **Work hours** |  |  |  |  |
| Sitting, min/10h | 12M | **-41.3 min (-63.2, -19.4), p<0.001** | **-38.0 min (-66.6, -9.5), p=0.009** | +3.3 min (-32.5, 39.2), p=0.856 |
| Prolonged sitting, min/10h | 12M | **-42.2 min (-72.0, -12.5), p=0.005** | **-46.2 min (-85.3, -7.1), p=0.021** | -4.0 min (-52.9, 45.0), p=0.874 |
| Time between sitting bouts | 12M | +1.8 min (0.0, 3.6), p=0.052 | +1.7 min (-0.6, 3.9), p=0.146 | -0.1 min (-2.9, 2.7), p=0.947 |
| Standing, min/10h | 12M | **+39.5 min (21.0, 58.0), p=0.015** | **+28.6 min (5.6, 51.6), p<0.001** | -10.9 min (-40.4, 18.5), p=0.467 |
| Stepping, min/10h | 12M | +2.6 min (-4.2, 9.3), p=0.452 | **+9.8 min (0.0, 19.5), p=0.049** | +7.2 min (-3.9, 18.3), p=0.203 |
| Number of steps/10h | 12M | +127.7 steps (-200.1, 455.5), p=0.444 | +468.2 steps (-23.8, 960.3), p=0.062 | +340.5 steps (-219.4, 900.4), p=0.232 |
| **Overall hours** |  |  |  |  |
| Sitting, min/16h | 12M | **-33.3 min (-63.6, -2.9), p=0.032** | **-41.2 min (-76.3, -6.1), p=0.022** | -7.9 min (-53.2, 37.3), p=0.732 |
| Prolonged sitting, min/16h | 12M | -31.0 min (-62.2, 0.3), p=0.052 | -24.9 min (-61.8, 11.9), p=0.185 | +6.1 min (-41.0, 53.2), p=0.800 |
| Time between sitting bouts | 12M | +1.1 min (-0.3, 2.5), p=0.137 | +1.7 min (-0.3, 3.8), p=0.099 | +0.6 min (-1.6, 2.9), p=0.571 |
| Standing, min/16h | 12M | **+33.9 min (11.3, 56.5), p=0.003** | **+30.4 min (2.0, 58.8), p=0.036** | -3.5 min (-39.2, 32.2), p=0.846 |
| Stepping, min/16h | 12M | -3.9 min (-15.5, 7.6), p=0.506 | +11.2 min (-2.5, 25.0), p=0.110 | +15.1 min (-2.5, 32.7), p=0.092 |
| Number of steps/16h | 12M | -138.7 steps (-679.3, 401.9), p=0.615 | +479.8 steps (-163.2, 1122.8), p=0.143 | +618.5 steps (-194.1, 1431.1), p=0.136 |

Significant changes (p<0.05) are in bold.
