# Supplementary material for: Evaluating the effectiveness of organisational-level strategies with or without an activity tracker to reduce office workers’ sitting time: a cluster-randomised trial
Source: Int J Behav Nutr Phys Act. 2016 Nov 4;13:115. doi: 10.1186/s12966-016-0441-3 (PMC5097432; doi:10.1186/s12966-016-0441-3)
Supplement: Additional file 8: — Part A: Uptake, baseline characteristics and activPAL completion by team. ​Part B: ICCs (95 % CI) for team clustering at baseline. (DOCX 27 kb) [file 12966_2016_441_MOESM8_ESM.docx]

Additional file 8.

Part A: Uptake, baseline characteristics and activPAL completion by team

| Group ORG teams | Team 1 | Team 3 | Team 6 | Team 8 | Team 9 | Team 12 | Team 14 | Team 16 | Team 18 |
| --- | --- | --- | --- | --- | --- | --- | --- | --- | --- |
| #Team members approached to participate | 8 | 9 | 11 | 16 | 14 | 15 | 17 | 13 | 14 |
| #Team members participating | 62.5% (5) | 88.9% (8) | 72.7% (8) | 81.3% (13) | 85.7% (12) | 53.3% (8) | 70.6% (12) | 61.5% (8) | 92.9% (13) |
| Male | 20.0% (1) | 62.5% (5) | 75.0% (6) | 30.8% (4) | 91.7% (11) | 75.0% (6) | 50.0% (6) | 37.5% (3) | 76.9% (10) |
| Age, years | 36.5±2.7 | 37.4±8.5 | 44.8±8.5 | 35.2±8.9 | 43.6±6.7 | 41.0±6.0 | 40.1±8.9 | 39.4±8.5 | 41.4±7.0 |
| BMI, kg/m^2^ | 23.8±2.8 | 24.5±2.2 | 26.8±2.1 | 22.5±2.9 | 24.6±2.0 | 25.6±3.5 | 24.3±4.4 | 26.9±4.4 | 26.6±3.5 |
| University education | 100.0% (4) | 100.0% (7) | 75.0% (6) | 84.6% (11) | 75.0% (9) | 50.0% (4) | 100.0% (11) | 75.0% (6) | 90% (9) |
| *Job category* |  |  |  |  |  |  |  |  |  |
| Manager | 60.0% (3) | 25.0% (2) | 62.5% (5) | 23.1% (3) | 41.7% (5) | 62.5% (5) | 83.3% (10) | 50.0% (4) | 61.5% (8) |
| Senior leader | 40.0% (2) | 37.5% (3) | 12.5% (1) | 7.7% (1) | 33.3% (4) | 12.5% (1) | - | - | 15.4% (2) |
| Other | - | 3.5% (3) | 25.0% (2) | 69.2% (9) | 25.0% (3) | 25.0% (2) | 16.7% (2) | 50.0% (4) | 23.1% (3) |
| **Activity variables at baseline** |  |  |  |  |  |  |  |  |  |
| Work hour sitting (min/10h workday) | 460.9±64.4 | 452.4±51.5 | 458.8±34.2 | 472.9±40.8 | 411.2±68.0 | 440.1±24.9 | 402.7±83.7 | 464.4±59.6 | 435.2±51.3 |
| Overall sitting (min/16h day) | 632.8±70.6 | 626.2±72.4 | 656.3±79.1 | 607.8±55.7 | 612.8±58.6 | 630.4±44.3 | 563.4±81.0 | 652.6±101.2 | 624.4±65.2 |
| **activPAL completion** |  |  |  |  |  |  |  |  |  |
| #3 months activPAL ^a^ | 4 | 7  (work = 6) | 6 | 13 | 9 | 7 | 10  (work = 9) | 4 | 8  (work = 7) |
| #12 months activPAL ^a^ | 2 | 5 | 2 | 7 | 6  (work = 5) | 3 | 6  (work = 5) | 3 | 4 |
|  |  |  |  |  |  |  |  |  |  |
|  |  |  |  |  |  |  |  |  |  |
|  |  |  |  |  |  |  |  |  |  |
|  |  |  |  |  |  |  |  |  |  |
|  |  |  |  |  |  |  |  |  |  |
|  |  |  |  |  |  |  |  |  |  |
| Group ORG+Tracker teams | Team 2 | Team 4 | Team 5 | Team 7 | Team 10 | Team 11 | Team 13 | Team 15 | Team 17 |
| #Team members approached to participate | 9 | 9 | 6 | 14 | 11 | 11 | 11 | 16 | 6 |
| #Team members participating | 88.9% (8) | 66.7% (6) | 50.0% (3) | 100.0% (14) | 81.8% (9) | 27.3% (3) | 72.7% (8) | 68.8% (11) | 66.7% (4) |
| Male | 25.0% (2) | 66.7% (4) | 33.3% (1) | 71.4% (10) | 33.3% (3) | 33.3% (1) | 37.5% (3) | 36.4% (4) | 75.0% (3) |
| Age, years | 41.5±10.0 | 30.4±4.3 | 37.7±3.2 | 37.1±6.7 | 37.9±8.7 | 41.0±8.2 | 36.6±6.0 | 39.3±9.7 | 34.5±5.9 |
| BMI, kg/m^2^ | 25.3±3.5 | 22.5±2.3 | 23.3±1.4 | 23.4±2.2 | 23.7±2.2 | 21.4±2.2 | 28.7±5.7 | 23.7±2.3 | 23.8±5.5 |
| University education | 87.5% (7) | 100.0% (5) | 100.0% (3) | 92.9% (13) | 87.5% (7) | 100.0% (3) | 100.0% (8) | 40.0% (4) | 100.0% (4) |
| *Job category* |  |  |  |  |  |  |  |  |  |
| Manager | 62.5% (5) | 83.3% (5) | 100.0% (3) | 64.3% (9) | 55.6% (5) | 100.0% (3) | 62.5% (5) | 27.3% (3) | 100.0% (4) |
| Senior leader | - | - | - | 7.1% (1) | 33.3% (3) | - | - | - | - |
| Other | 37.5% (3) | 16.7% (1) | - | 28.6% (4) | 11.1% (1) | - | 37.5% (3) | 72.7% (8) | - |
| **Activity variables at baseline** |  |  |  |  |  |  |  |  |  |
| Work hour sitting (min/10h workday) | 440.0±45.4 | 460.8±44.5 | 490.6±36.2 | 446.4±85.1 | 424.3±55.6 | 423.4±14.7 | 489.6±35 | 458.5±37.3 | 454.5±36.0 |
| Overall sitting (min/16h day) | 639.6±71.7 | 655.2±71.6 | 669.4±59.4 | 610.6±71.7 | 599.3±43.4 | 573.0±34.4 | 672.7±68.1 | 623.7±60.4 | 606.1±40.6 |
| **activPAL completion** |  |  |  |  |  |  |  |  |  |
| #3 months activPAL ^a^ | 7  (work = 6) | 2 | 2 | 10  (work = 8) | 4 | 2 | 7 | 6  (work = 5) | 1 |
| #12 months activPAL ^a^ | 3  (work = 2) | 2 | 1 | 7  (work = 6) | 0 | 1 | 4 | 4 | 1 |

Data are % (n) or mean±SD

^a^ Follow up numbers for activity during work hours are reported in parentheses if different from total

Teams 1 to 14 are from location A, teams 15 to 18 are from location B

There was missing data on body mass index (BMI) and education, percentages are out of those who responded

Part B: ICCs (95% CI) for team clustering at baseline (n=18 clusters; n=146 [work hours]; n=149 participants [overall hours])^a^

|  | ICC (95% CI) | n/cluster, mean (min, max) |
| --- | --- | --- |
| **Activity outcomes work hours** |  |  |
| Sitting, min/10h | 0.070 (0.013, 0.299) | 8.1 (3, 14) |
| Prolonged sitting, min/10h | 0.065 (0.010, 0.335) | 8.1 (3, 14) |
| Time between sitting bouts | 0.046 (0.005, 0.309) | 8.1 (3, 14) |
| Standing, min/10h | 0.054 (0.008, 0.300) | 8.1 (3, 14) |
| Stepping, min/10h | 0.053 (0.006, 0.349) | 8.1 (3, 14) |
| Steps/10h | 0.065 (0.009, 0.350) | 8.1 (3, 14) |
| **Activity outcomes overall hours** |  |  |
| Sitting, min/16h | 0.055 (0.007, 0.331) | 8.3 (3, 14) |
| Prolonged sitting, min/16h | 0.027 (0.001, 0.514) | 8.3 (3, 14) |
| Time between sitting bouts | 0.061 (0.010, 0.308) | 8.3 (3, 14) |
| Standing, min/16h | 0.064 (0.009, 0.335) | 8.3 (3, 14) |
| Stepping, min/16h | 0.057 (0.008, 0.316) | 8.3 (3, 14) |
| Steps/16h | 0.080 (0.016, 0.320) | 8.3 (3, 14) |

^a^ calculated in Stata from random intercept models, REML estimation.
