# Supplementary material for: Evaluating the effectiveness of organisational-level strategies with or without an activity tracker to reduce office workers’ sitting time: a cluster-randomised trial
Source: Int J Behav Nutr Phys Act. 2016 Nov 4;13:115. doi: 10.1186/s12966-016-0441-3 (PMC5097432; doi:10.1186/s12966-016-0441-3)
Supplement: Additional file 9: — Part A. Delivery and use of intervention components. Part B. Any use of promoted strategies. Part C. Any use of other strategies. (DOCX 18 kb) [file 12966_2016_441_MOESM9_ESM.docx]

Additional file 9.

Part A. Delivery and use of intervention components

| Intervention component | Number of participants |
| --- | --- |
| **Group ORG**  **Group ORG+Tracker**  Information booklet &  emails delivered: | n=151/153 sent information booklet and 5/5 emails  2 did not receive either because:   - 1 left organisation - 1 withdrawal |
|  |  |
| **Group ORG+Tracker**  LUMObacks delivered: | n=61/66  5 did not receive because:   - 1 left organisation - 2 ineligible (incompatible phone, not ambulatory) - 1 refusal - 1 unknown |
| Use of LUMOback at least once in first 3 months: | n=43/61  18 did not uptake because:   - 6 too busy to set up - 4 personal disruptions during study - 3 technical difficulties - 1 pregnancy - 4 unknown |
| Any use of LUMOback in study completers at 12 months: | n=0/25 |

Part B. Any use of promoted strategies (in the past month), at three months and 12 months vs. baseline.

|  | Time  3 months, n=98  12 months, n=51 | Baseline | Follow-up period  Fishers Exact test 2-tailed |
| --- | --- | --- | --- |
| Stood up during a meeting | 3M | 56.1% (55) | **74.5% (73), p=0.002** |
|  | 12M | 56.9% (29) | **78.4% (40), p=0.007** |
| Attended meeting where the chairperson indicated that it was acceptable to stand | 3M | 22.4% (22) | **35.7% (35), p=0.024** |
|  | 12M | 23.5% (12) | **52.9% (27), p=0.004** |
| Walking meeting | 3M | 21.4% (21) | **43.9% (43), p<0.001** |
|  | 12M | 27.5% (14) | **49.0% (25), p=0.027** |
| Stair use | 3M | 93.9% (92) | 95.9% (94), p=0.625 |
|  | 12M | 94.1% (48) | 90.2% (46), p=0.500 |

Any use = rarely, sometimes, often or very often vs. never. Data are % (n). Bold text indicates significant changes from baseline at p<0.05.

Part C. Any use of other strategies (in the past month), at three months and 12 months vs. baseline.

|  | Time  3 months, n=98  12 months, n=51 | Baseline | Follow-up period  Fishers Exact test 2-tailed |
| --- | --- | --- | --- |
| Stood up while on the phone | 3M | 76.5% (75) | 80.6% (79), p=0.454 |
|  | 12M | 80.4% (41) | 90.2% (46), p=0.180 |
| Walked to talk to colleague rather than emailing | 3M | 98.0% (96) | 98.0% (96), p=1.000 |
|  | 12M | 100.0% (51) | 98.0% (50), p=1.000 |
| Walked to printer further away from office | 3M | 62.2% (61) | 66.3% (65), p=0.585 |
|  | 12M | 64.7% (33) | 72.5% (37), p=0.503 |
| Walked to bathroom further away from office | 3M | 55.1% (54) | 64.3% (63), p=0.108 |
|  | 12M | 56.9% (29) | **76.5% (39), p=0.031** |
| Walked to a centrally located bin instead of own bin | 3M | 68.4% (67) | 75.5% (74), p=0.230 |
|  | 12M | 72.5% (37) | 76.5% (39), p=0.791 |
| Noticed signage | 3M | 21.4% (21) | **55.1% (54), p<0.001** |
|  | 12M | 19.6% (12) | **62.7% (32), p<0.001** |
| Used prompts at desk | 3M | 9.2% (9) | 14.3% (14), p=0.332 |
|  | 12M | 7.8% (4) | 17.6% (9), p=0.227 |
| Used computer break software | 3M | 3.1% (3) | **13.3% (13), p=0.006** |
|  | 12M | 3.9% (2) | 9.8% (5), p=0.375 |
| Eaten lunch away from desk | 3M | 93.9% (92) | 94.9% (93), p=1.000 |
|  | 12M | 92.2% (47) | 90.2% (46), p=1.000 |
| Gone for a walk during lunch | 3M | 77.6% (76) | 80.6% (79), p=0.607 |
|  | 12M | 84.3% (43) | 90.2% (46), p=0.508 |
| Cycled to work | 3M | 17.3% (17) | 15.3% (15), p=0.687 |
|  | 12M | 19.6% (10) | 13.7% (7), p=0.250 |
| Walked to work | 3M | 32.7% (32) | 36.7% (36), p=0.523 |
|  | 12M | 39.2% (20) | 33.3% (17), p=0.581 |
| Walked laps of office | 3M | 24.5% (24) | 31.6% (31), p=0.189 |
|  | 12M | 31.4% (16) | 37.3% (19), p=0.508 |
| Participated in group physical activity sessions during work hours | 3M | 24.5% (24) | 24.5% (24), p=1.000 |
|  | 12M | 25.5% (13) | 33.3% (17), p=0.503 |

Any use = rarely, sometimes, often or very often vs. never. Data are % (n). Bold text indicates significant changes from baseline at p<0.05.
